# Acute Management of Trimalleolar Fracture

**DOI:** 10.7759/cureus.12536

**Published:** 2021-01-06

**Authors:** Thor S Stead, Lauren H Pomerantz, Latha Ganti, Leoh Leon, Samyr Elbadri

**Affiliations:** 1 Emergency Medicine, Brown University, Warren Alpert Medical School, Providence, USA; 2 Medicine, University of Central Florida College of Medicine, Orlando, USA; 3 Emergency Medicine, Envision Physician Services, Plantation, USA; 4 Emergency Medicine, University of Central Florida College of Medicine, Orlando, USA; 5 Emergency Medicine, Ocala Regional Medical Center, Ocala, USA; 6 Emergency Medicine, HCA Healthcare Graduate Medical Education Consortium, Emergency Medicine Residency Program of Greater Orlando, Orlando, USA

**Keywords:** trimalleolar fracture

## Abstract

Extremity trauma is a common emergency department presentation. The authors report a case of an elderly woman who sustained a trimalleolar ankle fracture. Emergency department care includes stabilization of the fracture with a splint, with careful assessment of neurovascular status. Trimalleolar fractures are unstable and thus almost always will require surgical repair. This is true even for elderly patients and those with co-morbidities. Patients who do not get a surgical repair for these fractures are at risk for significant morbidity, including compartment syndrome, arthritis, malunion, and loss of mobility.

## Introduction

A trimalleolar fracture is a fracture of the three large bones that make up the ankle joint: the lateral, medial, and posterior malleoli. Accounting for about seven percent of ankle fractures seen in orthopedic units, it is the rarest type of ankle fracture behind open fracture [1]. It is also the most severe of the various ankle fractures, nearly always requiring prompt surgical reduction and internal fixation. The population of highest incidence is women aged 75-84 at primary risk from fall injury. Secondarily, young and middle-aged men are at risk from high-impact trauma [2]. 

The posterior malleolus, actually an extension of the distal tibia, proves to be the most complex part of this injury and is rarely seen as an isolated fracture [3]. Patients with larger posterior malleolar fragments exhibit poorer functional outcome [4]. Moreover, the relative size of the fragment as a percentage of the total articular surface is a criterion for surgical intervention (exact value varies and is dependent on the amount of displacement) [5]. Significant comorbidities include cause for weak bones such as osteoporosis or diabetes mellitus, a vascular disease, which can compromise the soft tissue envelope, and other physical conditions such as obesity and restricted movement, which can complicate post-surgical recovery [6, 7]. However, even patients exhibiting significant medical history with advanced age should be considered for surgery. A 2001 prospective study found that patients over age fifty-five who underwent open reduction and internal fixation had a significantly better functional outcome and ankle range of motion compared to those who simply had a closed reduction [8]. 

## Case presentation

A 73-year old female with a significant past medical history of congestive heart failure (CHF) status post biventricular pacemaker implantation, hypertension (HTN), lupus, and depression, presented to the emergency department with the chief complaint of left ankle pain after a fall. At the time of the fall, she was walking towards the stairs and accidentally tripped over her slippers, landing on her left ankle. She noted a history of falling several times in the past; however, this was the first time she recalled getting injured. After landing on the left ankle, she immediately experienced severe, sharp pain that was non-radiating but worsened by movement and weight-bearing. She denied hitting her head, loss of consciousness, experiencing chest pain, shortness of breath, palpitations, abdominal pain, nausea, and vomiting. While the patient takes multiple medications for CHF and HTN, her medication history was negative for any blood thinners.

Upon arrival to the emergency department, the patient’s vital signs were stable and within normal limits. Tachypnea, tachycardia, declining O_2_ saturation, and fevers were notably absent. Her physical exam was remarkable for an edematous, exquisitely tender left ankle that was warm to the touch. The dorsal pedis pulse was 3+ on bilateral lower extremities. Otherwise, the physical exam was unremarkable, and the patient was in no acute distress. She was awake, alert, and oriented, with heart rate and breath sounds within normal limits. Laboratory evaluation, including complete blood count, chemistries, and coagulation factors were unrevealing. An electrocardiogram was done to determine if the cause of the fall was an arrhythmia or heart block found normal intervals and a paced rhythm.

**Figure 1:**
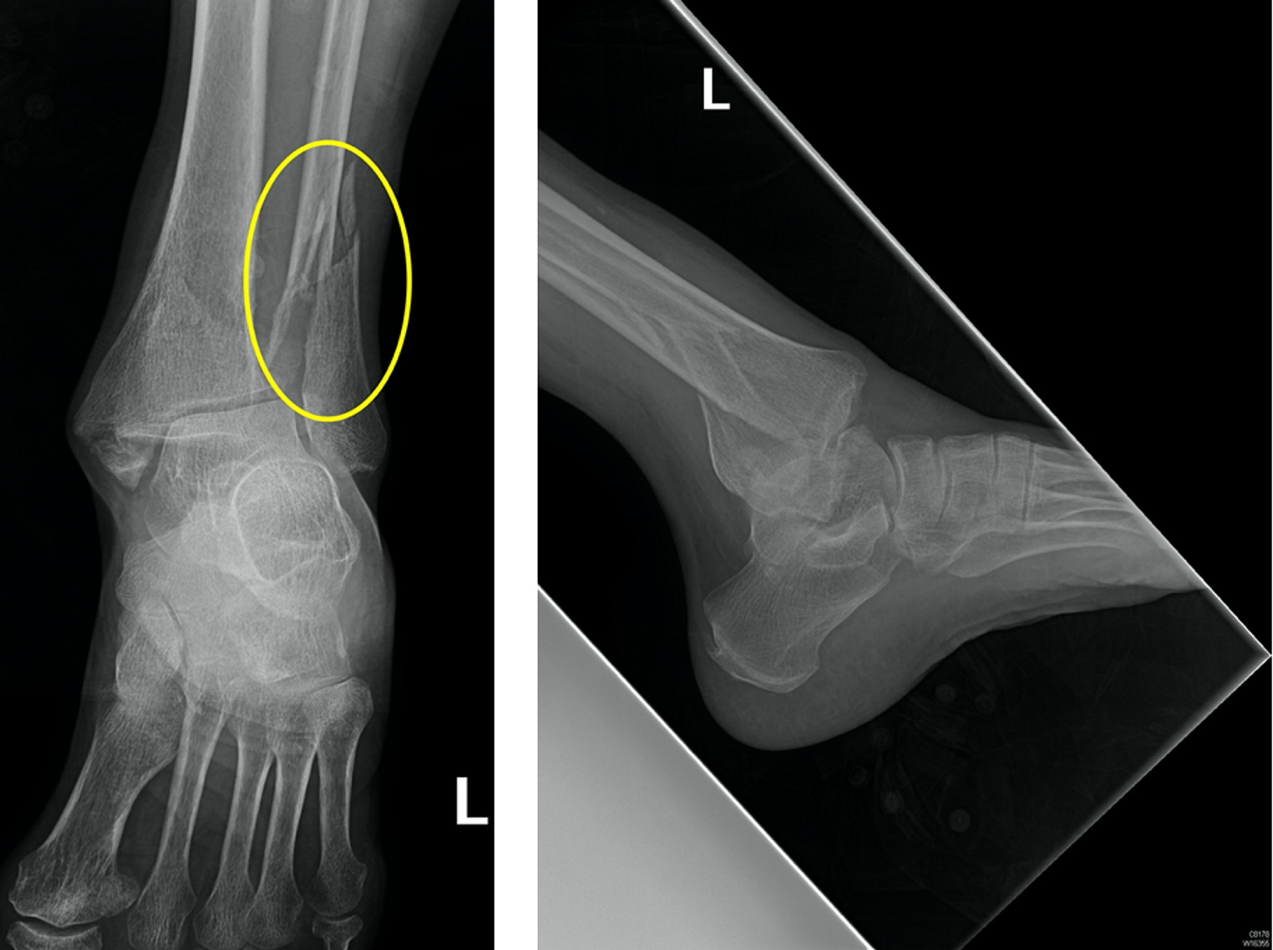
Pre-reduction anterior-posterior and lateral X-ray views demonstrating comminuted displaced trimalleolar fracture with a yellow circle highlighting the distal fibular fracture component

X-ray of the left ankle demonstrated a trimalleolar fracture dislocation. The talus was posteriorly dislocated in relation to the tibia (Figure 1).

**Figure 2:**
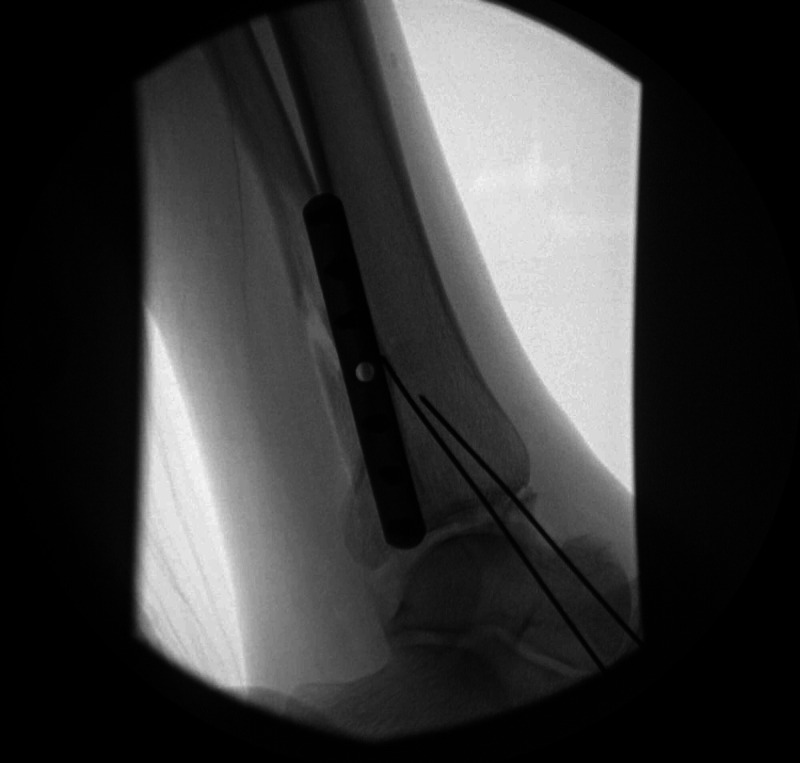
Post-reduction oblique fluoroscopic view visualizing the K-wires and locking plates with fracture displaying a proper reduction

The patient was consented for left ankle open reduction and internal fixation by the orthopedic team once all relevant data had been reviewed. She was given 2 mg midazolam before the procedure and 67 mg of ketamine during the procedure. After a 6 cm vertical incision was made over the fibula, and the fracture was visualized, a bone holding clamp significantly reduced the fracture. A 7-hole 1/3rd tubular plate in combination with locking and nonlocking screws was used for secure fixation to the bone. A second 2 cm vertical incision over the medial malleolus revealed that the posterior malleolus portion of the fracture encompassed about 10% of the joint, with the rest of the joint remaining stable. Posterior subluxation of the joint was absent. This part of the fracture was operatively repaired with two K-wires in the medial malleolus and two 3.0 mm partially threaded screws holding the malleolus in place. The wounds were irrigated, and fluoroscopy confirmed good placement of the hardware (Figure 2).

**Figure 3:**
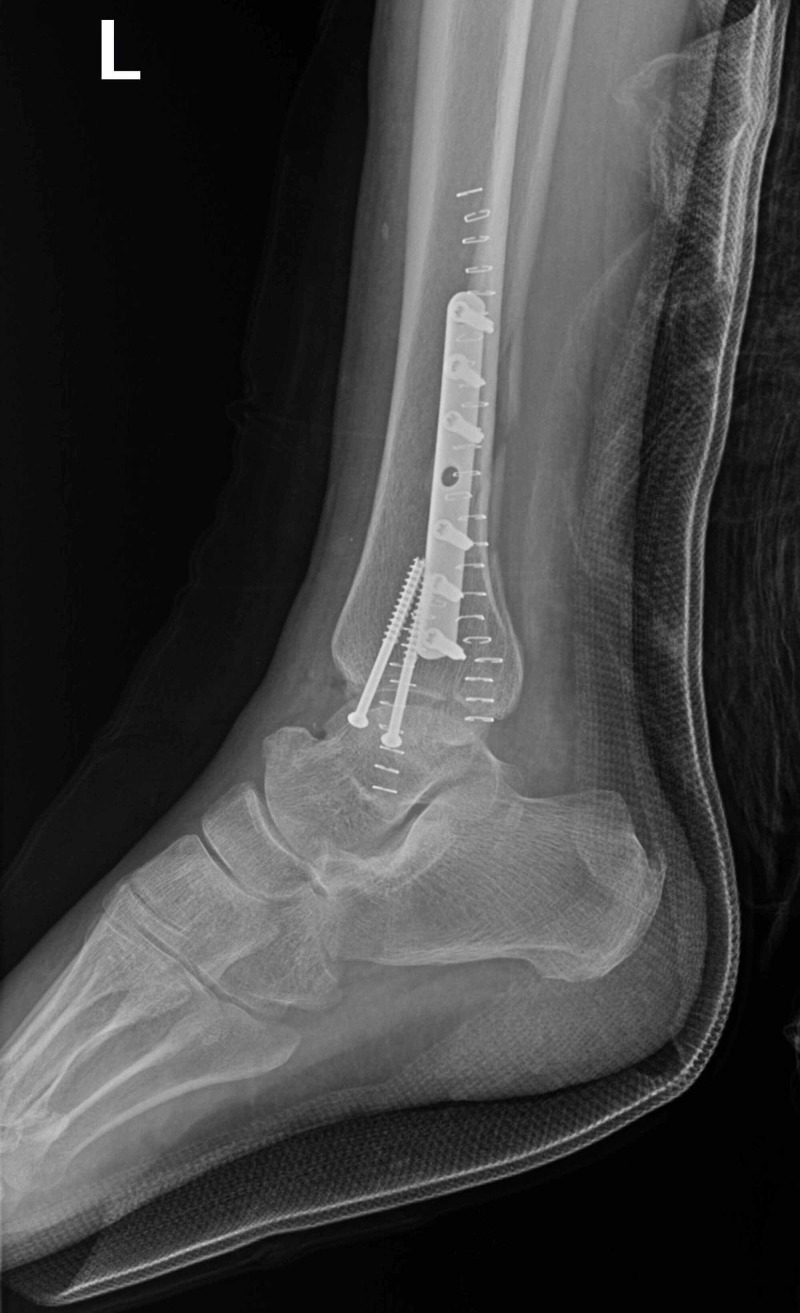
Post reduction lateral X-ray view visualizing complete repair and reduction of left ankle

On postoperative day 2, the patient transitioned to a controlled ankle motion walker boot and denied numbness, tingling, or other complaints. Ambulation was appropriate, and a follow-up X-ray revealed a successful reduction (Figure 3).

## Discussion

The ankle is an essential weight-bearing joint, and when multiple bones that make up this joint are fractured, the accurate reduction is of utmost importance. Many cases, such as the one illustrated in this report, only have minor posterior malleolar fragment fractures, with most of the ankle instability resulting from the lateral malleolus and the distal posterior fractured aspect of the tibia. A trimalleolar fracture is often at high risk of complication and typically requires surgical stabilization. They often occur in the setting of a high impact collision or fall. Unlike a twisting ankle injury that occurs due to an abnormal movement of the talus, a horizontal ankle fracture injury that occurs due to abnormal adduction or inversion, or a spiral ankle fracture that occurs due to an abnormal abduction or eversion of the ankle, the trimalleolar fracture typically results from an accident [9].

When a patient comes into the emergency department with a suspected ankle fracture, the key physical exam components that need to be inspected for are swelling, deformity, ability to bear weight, pulses, and ligament laxity. It is imperative that the clinician palpates the ankle to find the point of maximal tenderness and determine the ankle’s capacity for movement [10]. The pulses, as well as capillary refill, should be monitored to evaluate for fractures that might occlude the artery or compartment syndrome. Once the emergency conditions have been ruled out, the Ottawa ankle rules can ascertain if a radiograph is needed. According to the rules, ankle X-ray series is only required if there is a pain in the malleolar zone and bone tenderness at the posterior edge of lateral malleolus or medial malleolus or if there is an inability to bear weight for four steps [11]. Our patient clearly warranted imaging.

If a patient presents with a trimalleolar fracture and does not receive surgical correction, complications can ensue. Fractures that extend into the joint can promote loss of articular cartilage, which can progress to arthritis years later. If the fracture protrudes through the epidermal layer, promoting bacterial entry into the wound, then infection is also a risk. A prominent fracture that might pierce the layers of fascia, muscle, or skin may result in compartment syndrome. Finally, the trauma that created the trimalleolar fracture initially may also have been enough force to shear nerves or blood vessels to cause tears. If this were to occur, and surgery does not happen to correct the tears, permanent vascular or sensory loss might be at stake.

While this case utilized an open surgical approach, there are still various surgical approaches that can be taken when attempting an open reduction. The point of entry can be lateral, posterolateral, medial, or anterior, but each carries its own potential risk. The lateral approach is the most common, allowing for direct and complete visualization of the lateral malleolus, syndesmosis, and anterior and posterior aspects [10]. While this approach escapes concerns from internervous planes, the neurovascular structures at risk include the sural nerve, short saphenous vein, and peroneal artery that the surgeon must be aware of. The posterolateral approach utilizes the internervous plane between the flexor halluces longus. After appropriate retraction of the superficial peroneal nerve, the peroneal muscles and tendons may be displaced for access to the posterior malleolus. As long as the posterior tibial muscles and tibial nerves are protected, this method is an acceptable way to reach the distal posterior malleolus [10]. The medial approach is more common in a fracture surgery that requires subsequent osteochondral grafting of the talus. No internervous or intermuscular planes need to be dissected, and the medial malleolus, tibiotalar articular surface, and deltoid ligament can be visualized with ease. This medial approach is relatively safe regarding injury avoidance of neurovascular structures; however, the saphenous nerve and long saphenous vein might obstruct some views during retraction [10]. The anterior approach is mostly utilized in cases requiring access to the tibiotalar joint, such as a total ankle arthroplasty. The anterior approach carries the most risk of superficial cutaneous injury of the cutaneous superficial peroneal nerves, as well as the anterior tibial nerve. Ultimately, the open approach to ankle surgery allows the best opportunity for visualization when compared to an arthroscopic approach; however, caution and care have to be taken to avoid damaging at-risk structures - as with all surgery. Knowledge of ankle anatomy is imperative to efficiently avoid iatrogenic injury.

## Conclusions

Trimalleolar fractures are unstable ankle fractures. Surgical fixation results in the best outcomes and should be considered for all patients, regardless of co-morbidities. There are several approaches, with the anterior approach being most common. 
